# Family Accommodation and Separation Anxiety: The Moderating Role of Child Attachment

**DOI:** 10.21203/rs.3.rs-3621755/v1

**Published:** 2023-11-20

**Authors:** Gillian A. Weeks, Elcin Sakmar, Taylar A. Clark, Anastasia M. Rose, Wendy K. Silverman, Eli R. Lebowitz

**Affiliations:** Yale University School of Medicine

**Keywords:** separation anxiety, family accommodation, attachment, parenting, child

## Abstract

Family accommodation, or changes in parental behavior aimed at avoiding or alleviating child anxiety-related distress, contributes to the severity of anxiety symptoms, and is most strongly associated with separation anxiety symptoms. This study examined whether child attachment security, characterized as the degree to which children perceive their parents to be reliable, available, and communicative, moderates the association between family accommodation and separation anxiety symptoms, and whether this moderator is specific to separation anxiety among other anxiety symptoms. In a sample of clinically anxious youth (*N* = 243, 6–12 yrs), family accommodation was significantly positively associated with separation anxiety symptoms across levels of attachment security. Family accommodation was more strongly associated with separation anxiety symptoms in children with lower attachment security compared with those with higher attachment security. No significant moderation effect emerged for other anxiety symptoms. Findings enhance understanding of the role of attachment within family accommodation of child anxiety.

## Introduction

Anxiety disorders are the most common mental health problems among children (e.g., Costello et al., 2005; Polanczyk et al., 2015). Children with anxiety disorders experience impairment across academic, family, and social domains, and report lower life satisfaction compared with children without anxiety disorders (Swan & Kendall, 2016). Among the anxiety disorders, separation anxiety disorder is the most common for children under 12 years of age (American Psychiatric Association, 2013). Separation anxiety disorder is characterized by excessive and impairing concerns about being separated from attachment figures or home (American Psychiatric Association, 2013). Early in life, some anxiety upon separation from attachment figures is developmentally appropriate and evolutionarily adaptive (Feriante et al., 2023). In 4% of children, however, excessive separation anxiety persists into childhood and becomes impairing (Copeland et al., 2014; Shear et al., 2006). Symptoms present in a myriad of ways, including distress and/or physical symptoms upon separation, excessive concern that harm will befall their attachment figure or themselves when separated, refusal to sleep alone, and refusal to attend school, which contribute to significant impairment for the child and family (American Psychiatric Association, 2013).

### Family Accommodation

Children, like all mammals, are born ill-prepared for independent life and thus, rely on their parents for many aspects of survival, including safety when experiencing threat. When young children feel scared, they exhibit fear cues that alert their attachment figures to respond by providing protection, regulation, and comfort (Bowlby, 1973; 1982). This social aspect of the immature anxiety response is typically adaptive. However, among children with anxiety disorders, who experience chronic overactivation of their threat detection systems, there is overactivation of the social signaling of fear, which leads parents to implement extensive *accommodations* for their child’s anxiety (Lebowitz et al., 2013).

Family accommodation of child anxiety refers to changes in parental behavior aimed at avoiding or alleviating child anxiety-related distress (Lebowitz et al., 2013). Family accommodation is exceedingly common among parents of children with anxiety disorders, with an estimated 95–100% of parents reporting some degree of family accommodation (Benito et al., 2015; Lebowitz et al., 2013; Shimshoni et al., 2019; Thompson-Hollands et al., 2014). Accommodation for separation anxiety, particularly by parents, takes many forms, such as allowing a child to sleep in their bed, avoiding leaving the child with a babysitter or relative, and remaining present at playdates or birthday parties (Thompson-Hollands et al., 2014). Although well-intentioned, family accommodation plays a role in maintaining, rather than diminishing, child anxiety over time (Lebowitz et al., 2013). When parents accommodate a child’s anxiety, they unintentionally communicate that the child is unable to handle fear independently (Lebowitz et al., 2013; Norman et al., 2015; Shimshoni et al., 2019). This message increases child avoidance of anxiety-provoking stimuli, which contributes to more severe impairment in a child’s life (Swan & Kendall, 2016). Extensive empirical evidence supports this conceptual model whereby the degree of family accommodation is positively correlated with anxiety symptom severity and impairment (Benito et al., 2015; de Barros et al., 2020; Kagan et al., 2016; 2017; La Buissonnière-Ariza et al., 2018; Lebowitz et al., 2013; Lebowitz et al., 2014; Storch et al., 2015; Thompson-Holland et al., 2014).

#### Family Accommodation in Youth Separation Anxiety

Although family accommodation is prevalent across all anxiety disorders, studies have reported particularly high levels of family accommodation for separation anxiety symptoms (La Buissonnière-Ariza et al., 2018; Lebowitz et al., 2013; Storch et al., 2015; Thompson-Holland et al., 2014). Lebowitz and colleagues (2016) found biological evidence in support of this association, showing that salivary oxytocin levels in anxious children were significantly associated with degree of family accommodation and were most strongly associated with separation anxiety symptoms compared with other anxiety symptoms. Parents may be more willing to accommodate separation anxiety as their child’s desire for closeness with them may be viewed more positively than accommodations of other types of anxiety (Storch et al., 2015). The strong association between family accommodation and separation anxiety symptoms is also unsurprising given that separation anxiety is inherently related to the presence and behavior of attachment figures.

### Moderation of Family Accommodation and Youth Anxiety

As summarized above, family accommodation plays a key role in the course of child anxiety, but relatively little is known about factors that predict for which children accommodation may be particularly impactful. Several variables have been found though, that moderate the association between family accommodation levels and overall child anxiety symptoms. Settipani and Kendall (2017) found maternal anxiety and maternal empathy significantly moderated the link between child anxiety distress and family accommodation in clinically anxious youth (*N* = 70, 7–17 yrs). Schleider and colleagues (2018) found family accommodation was significantly associated with anxiety symptom severity in clinically anxious children with low anxiety sensitivity, but not children with high anxiety sensitivity (N = 103, 6–17 yrs).

#### The Role of Child-Parent Attachment

The particularly strong association between family accommodation and separation anxiety symptoms relative to other anxiety symptom subtypes suggests there may be unique moderators of this association. In considering separation anxiety, one variable that may moderate family accommodation is child attachment security. As theorized by Bowlby and illustrated by Mary Ainsworth in the seminal “strange situation” paradigm, securely attached children separate more easily and with less anxiety compared with insecurely attached children and recover more quickly than insecurely attached children upon reunification with their caregiver (Ainsworth, 1979; 1989). The positive internal working model of the caregiver as a “secure base” provides the basis for secure separation, without fear of abandonment (Ainsworth, 1979; 1989; Bowlby, 1973; 1982). Given that children differ in their ability to separate from their caregivers based on their attachment style, attachment security may play a more significant role for separation anxiety symptoms than for other anxiety symptom subtypes. In line with this theory, insecure attachment is associated with separation anxiety symptoms and insecure infant attachment predicts separation anxiety symptoms at age six (Brumarui & Kerns, 2010; Dallaire & Weinraub, 2005; Mofrad et al., 2010).

Family accommodation’s impact on separation anxiety may also vary based on a child’s attachment security, much like a child’s response to reunification in the strange situation varies based on attachment style. For example, in less securely attached children, a parents’ accommodation could serve to reinforce internalized feelings of vulnerability in a hostile and untrustworthy world, leading to more severe anxiety (Blandin et al., 1994). In more securely attached children, the parents’ accommodation may be less likely to exacerbate anxiety severity as it could be perceived as part of the internalized secure base the parent provides (De Wolff & Ijzendoorn, 1997; Koehn & Kerns, 2018; Nievar & Becker, 2008). No studies have yet to examine the potential interactions between family accommodation and attachment security in relation to child separation anxiety symptoms.

### Present Study

Family accommodation has been identified as an important factor in maintaining anxiety and is particularly strongly associated with symptoms of separation anxiety. Although both family accommodation and separation anxiety concern the child-parent attachment relationship, this study is the first to examine child attachment within the context of these two constructs. Understanding the role of child attachment security within this well-established association helps advance our conceptualization of separation anxiety and our understanding of for which children family accommodation is most important. The goal of the present study was to examine how child attachment security moderates the association between family accommodation and separation anxiety symptoms, and to determine whether this moderation effect is specific to separation anxiety symptoms among other anxiety symptom subtypes and overall anxiety symptoms. Family accommodation was hypothesized to be more strongly associated with separation anxiety symptoms in children with lower attachment security than children with higher attachment security.

## Method

### Participants

Participants were 243 children between 6 and 12 years of age (*M* = 8.26 years, *SD* = 1.67) and their mothers who were evaluated at an anxiety clinic in the United States. All children met for a clinical diagnosis of an anxiety disorder. Of the children, 154 (63.4%) met for a clinical diagnosis of separation anxiety disorder. Regarding children’s biological sex, 56.8% (*n* = 138) were male. Regarding children’s race, 78.6% were White, 2.9% were Black or African American, 2.5% were Asian American, 11.5% were multiracial, and 4.5% did not report race. Regarding ethnicity, 82.3% (*n* = 200) were not Hispanic or Latino. Mothers reported on family income, with 0.8% reporting less than $20,999, 4.1% between $21,000 and $40,999, 5.8% between $41,000 and $60,999, 8.6% between $61,000 and $80,999, 7.8% between $81,000 and $99,999, 23.1% between $100,000 and $149,000, 44.4% over $150,000, and 5.3% did not report income.

### Procedure

Mothers and their children attended an in-person visit as part of an intake evaluation for a clinical trial for anxiety treatment. Only data collected at baseline is used in the present study. Both mothers and children individually completed a battery of surveys including those listed below. Mothers also provided demographic information. All procedures were approved by the university’s Institutional Review Board.

### Measures

#### Screen for Child Anxiety Related Emotional Disorders, Parent Version (Birmaher et al., 1997)

The parent version of the Screen for Child Anxiety Related Emotional Disorders (SCARED) assesses a range of child anxiety symptoms over the past three months. Subscales include separation anxiety, generalized anxiety, social anxiety, panic, and school avoidance. The scale includes 41 statements such as “When my child feels frightened, it is hard for him/her to breathe,” answered on a three-point scale (0 = never true, 1 = sometimes true, 2 = very true), with higher scores indicating more anxiety symptoms. The SCARED has shown strong discriminant validity between children with and without anxiety disorders, and good internal consistency and test-retest reliability (Muris, 1998; 1999; Simon & Bogels, 2009). The Chronbach’s alpha for this sample was .89 for the total scale and ranged between .73 and .90 for the subscales.

#### Family Accommodation Scale–Anxiety (Lebowitz et al., 2013; 2015)

The Family Accommodation Scale–Anxiety (FASA) is a parent-reported 13-item scale that assesses the degree of parental involvement related to a child’s anxiety in the past month. The first nine questions measure frequency of family accommodation on a five-point scale from 0 = Never to 5 = Daily and are summed for a total accommodation score with higher scores indicating more frequent accommodation. Five of these questions measure family *participation* in anxiety symptom related behaviors (i.e., “How often did you reassure your child?”) and four measure *modification* of family life (i.e., “Have you modified your family routine because of your child’s symptoms?”). A single additional item measures the degree to which family accommodation causes the parent distress, and three items query short-term negative consequences of not accommodating, such as a child becoming angry. The FASA has demonstrated convergent and discriminant validity with measures of anxiety and depression respectively, good test-retest reliability, and high internal consistency (Lebowitz et al., 2013; 2020a). The Chronbach’s alpha for the nine-item total scale in the current sample was 0.85.

#### Kerns Security Scale (Kerns et al., 1996)

The Kerns Security Scale (KSS) assesses children’s perception of attachment security to their parents based on three subscales: *availability* (degree of parents’ responsiveness and availability), *reliance* (tendency of child to rely on parents in times of stress), and *communication* (interest and ease in communicating with parents). Fifteen questions are phrased in the format of “Some kids…” and “other kids…” and children are asked to select which one sounds more like them (i.e., “Some kids find it easy to trust their parents” and “Other kids are not sure if they can trust their parents”). After each question, children are asked “How much does this sound like you?” on a four-point scale from “Sort of True” to “Really True.” A score is calculated for each question based on which item was selected and how true it is. Some items were reverse coded, so that higher scores indicate greater attachment security. The KSS has demonstrated good internal consistency and high convergent validity with other measures of child attachment (Boldt et al., 2014; Brumariu et al., 2018; Kerns et al., 1996). The Chronbach’s alpha for the total scale in this sample was .76.

## Data Analysis

Data analyses were conducted using SPSS 28. First, data screening, including missing values analysis and confirming relevant statistical assumptions, was performed. All statistical test assumptions were met for multiple regression analyses. Little’s missing completely at random test’s (MCAR; 1988) results showed that the missingness pattern was completely random (*χ*2 (12) = 19.10, *p* = .086). Therefore, mean imputation was used to deal with missing data.

Spearman’s rho correlations were used to examine the associations between child attachment, family accommodation, and anxiety scales, including anxiety subscales. The moderating effect of child attachment security on the association between family accommodation and child separation anxiety symptoms was examined in a series of hierarchical multiple regression, with child separation anxiety symptoms as the dependent variable, controlling for child’s biological sex and age. Child attachment security and family accommodation were mean centered to reduce multicollinearity. In the first step, child separation anxiety symptoms were predicted from family accommodation and child attachment security while controlling for child’s biological sex and age. In the second step, the interaction term equal to the product of mean centered family accommodation and child attachment security was added to the model. Then, for the significant interaction, simple slopes were calculated for the association between family accommodation and child separation anxiety symptoms at lower (one standard deviation below the mean) and higher (one standard deviation above the mean) levels of child attachment security.

In addition to the main aim, focused on separation anxiety symptoms in particular, the analyses were also performed for predicting overall anxiety, generalized anxiety, social anxiety, panic, and school avoidance symptoms, separately.

## Results

Descriptive statistics and Spearman’s rho correlations of the main study variables are shown in Table 1.

### Moderation Analysis

**Figure 1:**
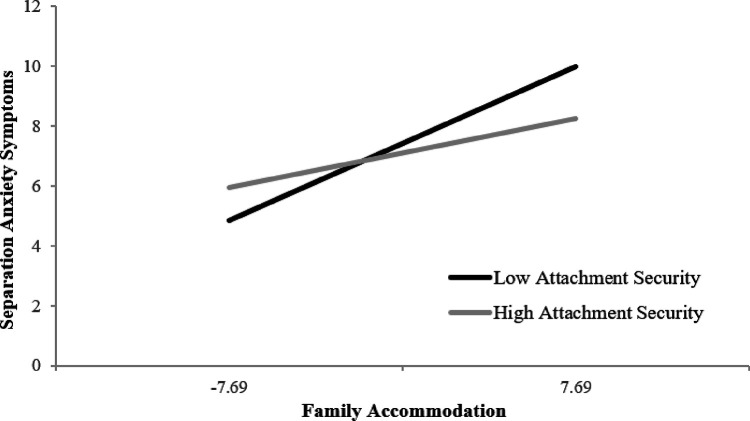
Association Between Family Accommodation and Separation Anxiety Symptoms

Results of the hierarchical multiple regressions with family accommodation and child attachment security as the independent variables and child separation anxiety symptoms as the dependent variable, controlling for biological sex and age, were indicative of moderation. The first step, including family accommodation and child attachment security, and controlling for child’s biological sex and age, resulted in an explained variance in child separation anxiety symptoms of *R*^2^ = .23 (adjusted *R*^2^ = .21) which was significantly different from zero (*F* (5, 238) = 17.49, *p* < .001). In the second step, there was a significant interaction effect between child attachment security and family accommodation in predicting separation anxiety symptoms (*R*^2^ = .25 (adjusted *R*^2^ = .24), *F*(5, 237) = 16.06, *p* < .001; B = −0.01, SE = 0.004, *p* = .005) (Table 2). With the addition of the interaction term equal to the product of family accommodation and child attachment, the explained variance in child separation anxiety symptoms increased by 2.6% which was significantly different from step one (ΔF(1, 237) = 8.21, *p* = .005). Post-hoc regression analyses of the interaction revealed that the positive association between family accommodation and separation anxiety symptoms was statistically significant at both lower (*B* = 0.33, *SE* = 0.05, *p* < .0001) and higher (*B* = 0.15, *SE* = 0.04, *p* = .0002) attachment security. However, a t-test demonstrated this association was significantly stronger for children who reported lower attachment security than for those who reported higher attachment security (*t*(237) = 2.94, *p* < .004). Figure 1 plots the simple slopes for the interactions.

Child attachment security did not significantly moderate the association between family accommodation and total anxiety (*B* = −0.02, *SE* = 0.013, *p* = .068), generalized anxiety (*B* = 0.00, *SE* = 0.005, *p* = .799), social anxiety (*B* = − 0.00, *SE* = 0.005, *p* = .692), panic (*B* = − 0.00, *SE* = 0.000, *p* = .348), or school avoidance symptoms (*B* = − 0.00, *SE* = 0.000, *p* = .080), while controlling for child’s biological sex and age.

## Discussion

These findings are the first to demonstrate the moderating role of child attachment security in the link between family accommodation and separation anxiety symptoms. In line with prior research, a positive and significant association was identified between family accommodation and separation anxiety symptoms for children with both lower and higher attachment security (Benito et al., 2015; Kagan et al., 2016; 2017; La Buissonnière-Ariza et al., 2018; Lebowitz et al., 2013; Lebowitz et al., 2014; Storch et al., 2015; Thompson-Holland et al., 2014). The strength of the association, however, was moderated by child attachment security, such that family accommodation was more strongly associated with separation anxiety symptoms in children with lower attachment security compared with those with higher attachment security. Notably, the moderation by attachment security was only apparent when predicting separation anxiety symptoms specifically. When the model was used to predict other anxiety symptom domains, or anxiety symptoms overall, no significant moderation emerged.

One possible explanation for the association between family accommodation and separation anxiety symptoms being weaker when child attachment security is higher is that a positive internal working model and perception of a secure base partially buffers the impact of family accommodation. When parents accommodate, they may be inadvertently communicating that they see the child as vulnerable or weak, which may be associated with greater separation anxiety in insecurely attached children. Another plausible way to understand this finding is that family accommodation may provide fewer opportunities for children to test maladaptive hypotheses surrounding their anxiety (Storch et al., 2007). Children with lower attachment security perceive their parents as less reliable than do children with higher attachment security and thus less certain to return after a separation. When parents accommodate their children’s separation anxiety by separating as infrequently as possible, these children may have limited opportunities to challenge this perception of their parents. Future longitudinal research should examine mediation models to explore these and other possible explanations.

### Clinical Implications

Interventions to improve attachment security in young children (e.g., Attachment and Biobehavioral Catch-Up and Video-feedback Intervention to promote Positive Parenting and Sensitive Discipline) have been found to be efficacious and to have a variety of beneficial effects for children (Van Ijzendoorn et al., 2023; Zajac et al., 2020). Findings demonstrating that family accommodation is less predictive of separation anxiety symptoms for children with higher attachment security suggest a potential additional benefit of these kinds of interventions.

Findings linking greater family accommodation to more severe separation anxiety symptoms underscore the importance of parent-based interventions targeting family accommodation in the treatment of separation anxiety. SPACE (Supportive Parenting for Anxious Childhood Emotions) is one such parent-based intervention that focuses heavily on accommodation reduction and has been found to be efficacious (Lebowitz et al., 2020b). The finding that family accommodation is most strongly predictive of separation anxiety symptoms when attachment security is low suggests that less securely attached children with separation anxiety may stand to particularly benefit from this kind of intervention, as opposed to other types of treatment.

### Limitations and Future Directions

The conclusions that can be drawn from this study are limited by the study’s cross-sectional design. It is not possible to draw causal inferences from the cross-sectional data and additional research is required to understand the causal pathways linking family accommodation and separation anxiety symptoms as well as the role of attachment security. Clinical trial research is required to investigate whether attachment interventions can reduce separation anxiety symptoms when family accommodation is present and whether accommodation-reducing parent-based interventions for separation anxiety are more efficacious for children with lower attachment security than for children with higher attachment security.

This study is further limited in that mother’s report of child attachment security was not collected. However, shared reporter variance was limited due to the use of mothers’ ratings to assess child anxiety symptoms and family accommodation, and children’s ratings to assess attachment security (Burke & Laursen, 2010). Additionally, no behavioral observations were used to examine family accommodation, anxiety symptoms, or attachment security. Future studies should extend this work by examining both mother and child report and behavioral observations.

In addition, this study is limited by a homogenous sample, comprising predominantly white, middle-upper class families. While much of research in youth anxiety disorders lacks sufficient diversity of samples and has been conducted with majority white, affluent participants (Benito et al., 2015; Storch et al., 2015), it is important to consider that findings may not generalize to broader populations. Family accommodation may differ in prevalence or form in minority groups and among low-income families, where socioeconomic stressors may impact how, and how much, parents accommodate. Future work should strive to confirm the findings of this study in more racially, ethnically, and socioeconomically diverse samples.

## Conclusion

Results support a model whereby children’s attachment security moderates the association between family accommodation and separation anxiety symptoms. This study is the first to demonstrate that family accommodation is more strongly associated with separation anxiety symptoms for children with lower attachment security than for children with higher attachment security. No such moderation effect was found for other anxiety symptom subtypes. Findings enhance understanding of the role of child attachment within family accommodation of youth anxiety.

## Figures and Tables

**Table 1:** Descriptive Statistics and Spearman’s Rho Correlations

	1	2	3	4	5	6	7	8
**1.KSS Total**	-							
**2. FASA**	.01	-						
**3. SCARED Total**	− .01	.48*	-					
**4. SCARED SP**	− .03	.44*	.69*	-				
**5. SCARED GD**	− .00	.29*	.72*	.36*	-			
**6. SCARED SO**	.03	.32*	.67*	.31*	.29*	-		
**7. SCARED PN**	− .02	.24*	.65*	.34*	.41*	.24*	-	
**8. SCARED SC**	.02	.34*	.54*	.29*	.27*	.30*	.27*	-
**Mean (SD)**	48.52 (7.48)	17.30 (7.69)	31.12 (12.33)	7.26 (4.10)	9.86 (4.07)	7.41 (4.13)	4.26 (3.73)	2.32 (2.10)

Note.

* *p* < .01; SP = Separation Anxiety, GD = Generalized Anxiety, SO = Social Anxiety, PN = Panic, SC = School Avoidance

**Table 2:** Results of Moderation Analysis

	*B*	*SE*	*t*	*P*
Constant	10.58	1.24	8.53	*p* < .001
FASA (centered)	0.24	0.03	7.99	*p* < .001
KSS (centered)	−0.02	0.03	−0.69	*p* = .490
FASA (centered) * KSS (centered)	−0.01	0.004	−2.87	*p* = .005
Age	−0.42	0.14	−2.96	*p* = .003
Biological Sex	0.38	0.47	0.80	*p* = .424
